# Interplay Between Keratinocytes and Fibroblasts: A Systematic Review Providing a New Angle for Understanding Skin Fibrotic Disorders

**DOI:** 10.3389/fimmu.2020.00648

**Published:** 2020-05-06

**Authors:** Barbara Russo, Nicolò C. Brembilla, Carlo Chizzolini

**Affiliations:** ^1^Department of Pathology and Immunology, School of Medicine, University of Geneva, Geneva, Switzerland; ^2^Dermatology, School of Medicine, University Hospital, Geneva, Switzerland

**Keywords:** fibrosis, keratinocyte, fibroblast, systemic sclerosis, cytokine, extracellular matrix, homeostasis

## Abstract

**Background/Objective:** Skin fibrosis is the result of aberrant processes leading to abnormal deposition of extracellular matrix (ECM) in the dermis. In healthy skin, keratinocytes participate to maintain skin homeostasis by actively crosstalking with fibroblasts. Within the wide spectrum of fibrotic skin disorders, relatively little attention has been devoted to the role of keratinocytes for their capacity to participate to skin fibrosis. This systematic review aims at summarizing the available knowledge on the reciprocal interplay of keratinocytes with fibroblasts and their soluble mediators in physiological states, mostly wound healing, and conditions associated with skin fibrosis.

**Methods:** We performed a systematic literature search on PubMed to identify *in vitro and ex vivo* human studies investigating the keratinocyte characteristics and their interplay with fibroblasts in physiological conditions and within fibrotic skin disorders including hypertrophic scars, keloids, and systemic sclerosis. Studies were selected according to pre-specified eligibility criteria. Data on study methods, models, stimuli and outcomes were retrieved and summarized according to pre-specified criteria.

**Results:** Among the 6,271 abstracts retrieved, 73 articles were included, of which 14 were specifically dealing with fibrotic skin pathologies. Fifty-six studies investigated how keratinocyte may affect fibroblast responses in terms of ECM-related genes or protein production, phenotype modification, and cytokine production. Most studies in both physiological conditions and fibrosis demonstrated that keratinocytes stimulate fibroblasts through the production of interleukin 1, inducing keratinocyte growth factor (KGF) and metalloproteinases in the fibroblasts. When the potential of keratinocytes to modulate collagen synthesis by healthy fibroblasts was explored, the results were controversial. Nevertheless, studies investigating keratinocytes from fibrotic skin, including keloids, hypertrophic scar, and scleroderma, suggested their potential involvement in enhancing ECM deposition. Twenty-three papers investigated keratinocyte proliferation differentiation and production of soluble mediators in response to interactions with fibroblasts. Most studies showed that fibroblasts modulate keratinocyte viability, proliferation, and differentiation. The production of KGF by fibroblast was identified as key for these functions.

**Conclusions:** This review condenses evidence for the active interaction between keratinocytes and fibroblasts in maintaining skin homeostasis and the altered homeostatic interplay between keratinocytes and dermal fibroblasts in scleroderma and scleroderma-like disorders.

## Introduction

Fibrosis is a complex process characterized by abnormal deposition of extracellular matrix (ECM), which can lead to altered tissue architecture impacting organ function and survival (1). Fibroblasts are endowed with the full machinery allowing deposition and resorption of ECM, which under homeostatic conditions is continually renewed. Fibroblast synthetic and degradative capacities are modulated by a variety of stimuli, which include soluble factors, cell-to-cell interactions, matrix stiffness, and tensile forces, oxygen levels, epigenetic changes, cell aging, telomere length, and cell survival (2).

Most importantly, fibroblasts are under the influence of a variety of other cell types, which are specifically resident in the tissue undergoing fibrotic changes or professional inflammatory cells recruited in the tissue (3). Soluble mediators of inflammation and, in particular, cytokines and growth factors are deeply involved in regulating fibroblast migration, proliferation, metabolism, and ECM deposition (4). In particular, TGF-β is considered a master mediator of fibrosis (5) relevant for the recruitment and trans-differentiation of cell precursors into myofibroblasts. These are cells with contractile properties associated with the expression of α-smooth actin and with a very high capacity to synthetize and release ECM components such as type I and type III collagen, fibronectin, and tenascin among others (6–8).

Enhanced ECM deposition is physiologically important and part of the reparative process in damaged tissues. Tissue damage can result from infectious agent assaults, or traumatic wounds, or the effect of physico-chemical injuries. Thus, enhanced ECM deposition is part of normal reparative inflammatory processes, and the characterization of wound healing has historically been fundamental to understand processes leading to fibrosis. What distinguishes controlled ECM enhanced deposition from pathological fibrosis is that the many mechanisms, which are important to halt ECM deposition, are relatively deficient to oppose persistent stimulation (1). Thus, perturbed homeostasis resulting from a variety of origins may explain excessive ECM deposition and pathological tissue fibrosis.

The skin is a tissue that can undergo fibrosis in response to local stimuli but also, while more rarely, as a result of systemic inflammatory disorders. Systemic sclerosis (SSc) or scleroderma is a prototypic condition in which dysregulated inflammation associated with autoimmunity and widespread vascular dysfunction results in skin and internal organs' pathological fibrosis (9). Localized skin fibrosis is observed in Morphea, hypertrophic scars, keloids, and many other conditions with metabolic, vascular, or genetic origins (10). Since long, it is known that the traumatic loss of epithelial cells (keratinocytes) and the following process of re-epithelization are spatially and chronologically important events regulating fibroblast activation and ECM deposition (wound healing) (11). More recent and less developed is our understanding of the role of keratinocytes for their capacity to regulate ECM deposition in non traumatic skin fibrosis. Similarly, relatively little is known about the role of fibroblasts and ECM for their influence on keratinocyte proliferation, differentiation, and epidermis generation. The present work aims to systematically review published evidence on the reciprocal role of keratinocytes and fibroblasts and their soluble products under the angle of human skin fibrosis.

## Methods

### Literature Search

We searched the literature on PubMed up to, and including, August 31, 2019. We conducted our search using a combination of free terms and controlled vocabulary terms by Boolean operators (AND, OR). The terms used were: [“myofibroblast” (Mesh) OR “mesangial cells” (Mesh) OR “fibroblast^*^” (tiab) OR “fibro^*^” (tiab)] AND (“dermis” (Mesh) OR “derm^*^” (tiab) OR “skin” (Mesh) OR “cutis^*^” (tiab) OR “cutan^*^” (tiab) OR “epithel^*^” (tiab) OR “keratin^*^” (tiab)]. Keywords were detected in titles and abstracts. We also reviewed reference lists of the included full text and of other reviews on the topic to find additional reports. The systematic review was performed according to the PRISMA guidelines (12).

### Inclusion Criteria

Studies fulfilling the following inclusion criteria were included in the present review: *in vitro* or *ex vivo* studies on cells or tissues of human origin from healthy donors or individuals affected by fibrotic pathologies with the exclusion of tumors or cancers. The focus was put on the keratinocyte–fibroblast interactions and the methods used to investigate these interactions, with no restrictions.

### Exclusion Criteria

We excluded studies on animals, animal tissues, animal cells, and animal pathologies. We excluded human studies on hair follicles, neoplastic conditions or neoplastic cell lines, as well as inflammatory skin pathologies with no evident fibrotic component. We excluded reviews and commentaries. We excluded studies not describing keratinocyte–fibroblast interactions. We excluded studies when the full text was not available and when the language was other than English.

### Data Extraction

We used standardized data extraction forms. For each study, the following items were collected: first author, year of publication, type of experimental models and methods used for investigating keratinocyte–fibroblast interactions, type of culture medium, type of stimuli and their outcomes, and mediators potentially responsible for the observed effect. Initially, titles and abstracts of all identified citations were reviewed. Full text of potentially relevant articles was screened and checked for eligibility. Disagreements about the inclusion of articles were resolved by two of the authors (BR, CC). In detail, abstract and full texts were reviewed together by the authors to reach a shared decision in case of disagreement.

### Summarizing and Interpreting the Data

Data were subdivided according to the main objective of the identified studies in two categories: studies reporting mainly the effects of keratinocytes on fibroblasts (Table 1), studies reporting mainly the effects of fibroblasts on keratinocytes (Table 2). Studies specifically addressing fibrotic skin disorders are summarized in Table 3. Reporting was focused on cell proliferation, differentiation, and migration, extracellular matrix components, and turnover, identification of soluble factors of inflammation and growth factors, skin pathology, type of activating stimuli. The studies describing reciprocal effects on both cells types were listed in both categories and tables.

**Table 1:** *In vitro* and *ex vivo* studies on the effect of human keratinocytes on dermal fibroblasts.

**Ref**	**Type of cells or samples**	**Type of stimuli**	**Identified mediators**	**Experimental outcome**
Dufour et al. (13)	NEK, HDF, SScF; K-CM; HD full skin explant	TGF-β, IL-17A	IL-1; TGF-β	Keratinocytes enhance IL-6, IL-8, and MCP-1, production by HDF and SScF. Keratinocytes enhance ECM turnover by enhancing MMP-1 and decreasing col-I. IL-17A increases these effects TGF-β reduces these effects
Fernando et al. (14)	HaCaT, HDF; HaCaT-CM	Particulate matter (PM) fucosterol		Increase of inflammatory responses (TNFα, IL-1β, IL-6, MMP1, MMP2, elastase, PGE2) in fibroblasts treated with media from HaCaT exposed to CPM. Fucosterol reduced these effects
Zhao et al. (15)	HaCaT; HDF; EE Transwell coculture Hypertrophic scar biopsies	Dehydration		HaCaT dehydration increases col-I and αSMA expression by HDF. HMGB1 KO in HaCaT decreases HDF activation induced by dehydration. Cytoplasm accumulation of HMGB1 in hypertophic scar
McCoy et al. (16)	SScK, NEK; HDF; K-CM		Not-TGF-β	SScK more than NEK enhance col-I and αSMA expression by HDF Microarray data on differences between SScK and NEK
Carr et al. (17)	NEK, HDF, HaCaT Transwell coculture K-CM	Differentiated and undifferentiated NEK	IL-1	NEK enhances G-CSF production by HDF Undifferentiated NEK have stronger effect than differentiated NEK
Brembilla et al. (18)	NEK, HDF, SScF K-CM	IL-22, TNFα		NEK and SScK promote HDF production of MMP-1, MCP-1, and IL-8. IL22 + TNFα enhances this effect
Zhong et al. (19)	HaCaT, foreskin K, foreskin F; Differentiated-K Transwell coculture Epidermal explant HD, keloids, hypertrophic scars biopsies	Low humidity/reduced hydration	S100A8/A9	S100A8/A9 is more expressed in epidermis from keloids and hypertrophic scars than HD HaCaT dehydration increases col-I and αSMA expression by HDF. Effect mediated by epidermal S100A8/A9, which expression is induced by reduced hydration
Huang et al. (20)	HaCaT, foreskin K, HDF Keratinocyte-derived microvesicles (K-MV)		Keratinocyte-derived microvesicles (K-MV)	K-MV enhance in HDF the expression of TGF-β-induced genes and of MMP-1, MMP-3, THBS1, IL-6, lumican; enhance HDF migration and matrix contraction, enhance HDF-dependent angiogenesis Decrease in HDF the expression of cadherin-2
Gauglitz et al. (21)	HDF keloid and normal skin biopsies 2D-culture		S100A7 S100A15	Compared to healthy skin reduced expression of S100A7 and S100A15 in keloids epidermis with reciprocal expression of COL1A1, COL1A2, COL3A1. S100A7 and S100A15 on HDF decrease COL1A1, COL1A2 and COL3A1, TGF-β1, TGF-β2, TGF-β3, laminin-β2 and α-SMA and HDF proliferation
Xu et al. (22)	HaCaT, foreskin k, foreskin F; Differentiated-K Transwell coculture Epidermal explants	Reduced hydration	ENaC, COX2, PGE2	HaCaT dehydration increases col-I and αSMA expression by HDF. Effects mediated by ENaC, COX2, PGE2
Arai et al. (23)	Foreskin-K, foreskin-F; EE DE (decellularized dermis) Skin equivalent EE-CM		IL-1α, IL-1β	PGE2 detected only in skin equivalent. PGE2 expressed by DD enhances keratinocytes proliferation EE-CM increases COX2, IL-6, and GM-CSF and decrease KGF expression in HDF
Nikitorowicz-Buniak et al. (24)	HD and SSc skin, HDF, SScF; HD, SSc epidermal and dermal explants; HD, SSc epidermal explant CM	S100A9	S100A9	Increase of CCN2, S100A9, HGF in SSc epidermis compared to dermis and HD epidermis; S100A9 enhances HDF and SScF proliferation, migration, and CTGF production
Li et al. (25)	HaCaT, HDF HaCaT-CM		Fibronectin	HaCaT–CM enhances HDF migration
Varkey et al. (26)	NEK; HDF EE DD (superficial (S)/or deep(D) HDF embedded in a GAG matrix) Skin equivalent			In organotypic cultures, the levels of col-I and fibronectin were lower and levels of TGFα, PDGF, IL-1 higher compared to embedded HDF only D-HDF produced higher levels of col-I higher levels of TGF-β activity and IL-6 compared to S-HDF S-HDF produced higher MMP-1 levels
Sun et al. (27)	HaCaT, fibroblast cell line CCD966SK 2D-culture		KGF, IL-19	IL-19 induces KGF expression in CCD966SK fibroblasts KGF enhances the production of IL-19 in HaCaT and promotes higher proliferation and migration
Canady et al. (28)	NEK, HDF, SScF, keloid fibroblasts, HD skin skin explant		KGF, OSM	KGF is increased in keloid and SSc fibroblasts and sera KGF induces keratinocytes to release OSM leading to fibroblast activation KGF increases the production of OSM, (fibroblast activator protein) FAP, col-I in cultured skin explants
Kolar et al. (29)	HaCaT, NEK, HDF EE; DE (collagen embedded HDF) Skin equivalent		IL-6, IL-8, CXCL-1	NEK-organotypic cultures enhance the expression of FGF-7, FGF-5, FGF-2, CXCL-1, IL-6, IL-8 in HDF
Rock et al. (30)	Female NEK and HDF K-CM		E2; E2 and UVB	E2 and E2 + UVB increase the production of EGF in NEK Conditioned medium from E2 and E2 + UVB-exposed KCM enhances hyaluronan synthase 3 and versican V2 and proliferation of HDF
Simon et al. (31)	K-from hypertrophic scars, NEK, HDF, hypertrophic scar F EE or EE-CM DE (F in a dermal matrix) Skin equivalent		TIMP-1	Compared to NEK, K from hypertrophic scars increase dermal matrix thickness, by enhanced production of TIMP-1
Do et al. (32)	Keloids-K, Keloid-F, NEK, HD Transwell coculture		IL-18	K form keloids more than NEK produce IL-18, fibroblasts from keloids and HDF enhance IL-18 production by keratinocytes IL-18 enhances col-I, IL-6, IL-8 production by HDF
Lai et al. (33)	NEK, HDF K-CM		Stratifin	Conditioned medium from NEK enhances the production by HDF of MMP-1, MMP-3, MMP-12, versican, TN-C, ITGA1, CTNNA1, FN NEK induce the upregulation of aminopeptidase N/CD13 in HDF as consequence of stratifin production
Tandara and Mustoe (34)	NEK; HDF Transwell coculture K-CM			K-CM enhance the production of MMP-1, MMP-8, MMP-13, MMP-2, MMP-10, TIMP-1, and TIMP-2 by HDF. NEK-hydration further increases the upregulation of MMPs and decreases TIMP-2
Koskela et al. (35)	NEK, HDF, EE DE (HDF embedded in collagen) Skin equivalent	TGF-β		Compared to HDF alone, organotypic cocultures increase MMP-1, MMP-3, uPA and decrease CTGF, col I, col III, FN, TIMP-2, αSMA, PAI, in the presence or absence of TGF-β
Aden et al. (36)	SSc and HD skin biopsies, HDF, SScF, SSc or HD epidermis explant DE (HDF embedded on collagen) Skin equivalent		IL-1, TGF-β, ET-1	Altered keratinocyte differentiation in SSc biopsies Compared to HD, SSc epidermal explants produce more IL-α resulting in enhanced gel contraction SSc and HD explants have similar levels of ET-1 or TGFβ. ET-1 and TGFβ have a role in CTGF production by HDF
Lim et al. (37)	Keloids-K, keloids-F, NEK, HDF. Monolayer, Transwell coculture			IL-6, IL-8, MCP1, TIMP-1, TIMP-2 detected in monocultures Angiogenin, OSM, VEGF, IGF-binding protein-1, OPG, and TGF-β2 detected in keloids-K-keloids-F, but absent in NEK–HDF cocultures
Chavez-Munoz et al. (38)	Differentiated and undifferentiated foreskin K, HDF K-Exosomes		14-3-3 (stratifin)	Exososomes generated from differentiated more than undifferentiated foreskin K enhance MMP-1 production by HDF. This effect is mediated by stratifin
Ghaffari et al. (39)	NEK, HDF Transwell coculture		keratinocyte-derived collagen-inhibitory factor of 30–50 kD (KD-CIF)	Keratinocyte-released factors reduce col-I production by HDF by KD-CIF Keratinocyte differentiation do not alter synthesis, release, or activity of KD-CIF
Wall et al. (40)	NEK, HDF DE (HDF) embedded in collagen gel Skin equivalent			In comparison to HDF cultured in monolayers, the production of MMP2, MMP9, uPA, uPAR is increased in organotypic cocultures, with no significant changes in contractile responses
Tandara et al. (41)	NEK, HDF Transwell coculture			Compared to HDF cultured in monolayers, col-I production is decreased, and KGF production increased in Transwell cultures, more so in hydrated cultures. Compared to NEK cultured in monolayers, TNF production is increased and IL-1 is decreased in Transwell cultures
Amjad et al. (42)	NEK, HDF, K-CM DE (HDF collagen embedded) Skin equivalent			NEK decrease TGFβ1 the production by HDF
Harrison et al. (43)	NEK, HDF Coculture			NEK conditioned medium and NEK coculture inhibit spontaneously, and IGF, bFGF-stimulated col-I production by HDF, TNF reduce this inhibition
Ghaffari et al. (44)	NEK, HDF Transwell coculture		Stratifin	Stratifin is produced only by NEK Stratifin and NEK-conditioned medium enhance MMPs, adhesion molecules, PAI1. PAI2, THSP1, FN (and other detected by microarray) by HDF
Harrison et al. (45)	NEK, HDF HD epidermal explants Coculture K-CM			Both NEK-conditioned medium and HD epidermal explants decrease HDF proliferation HD epidermal explants but not NEK-conditioned medium enhance FN production by HDF
Chinnathambi and Bickenbach (46)	NEK and HDF EE; DE (HDF collagen embedded) skin equivalent			Compared to HDF cultured in monolayers, the production of MMP1 is increased and MMP-2 is decreased in organotypic cocultures
Ghahary et al. (47)	NEK, HDF Transwell coculture		Stratifin	Compared to HDF cultured in monolayer, MMP1 is increased Stratifin induces MMP-1 Stratifin expression is higher in differentiated NEK
Sawicki et al. (48)	K-foreskin, HDF Transwell coculture			Compared to NEK cultured alone, HDF enhance the production of MMP-9 and MMP-2 by K. HDF cocultured with K produce MMP-9 TIMP-1, TIMP-2, and TIMP-3, but not, TIMP-4 levels are enhanced both in K and HDF when in coculture
Shephard et al. (49)	HaCaT, irradiated HDF Coculture			Compared to HDF cultured in monolayer, the contractile activity and αSMA expression is increased in coculture ET-1 enhances contraction and TGF-β enhances αSMA expression in cocultures
Shephard et al. (50)	HaCaT, NEK, irradiated and not irradiated HDF Coculture			Compared to HDF cultured alone, HDF in cocultures with HaCaT and NEK expresses more—*ENA-78*, and *MCP-1, IL-6, LIF, G-CSF, M-CSF, COX2*, PAI, and less *Cathepsin K, Cathepsin L, Cathepsin L2* *More col-I, col-IV, col-V, col-VI, hyaluran synthtease, lysine hydroxylase, transglutaminase 2, TN-C, decorin, syndecan 2, but less testican, tenascin XA, fibulin, thrombospondin* *a* SMA expression requires close proximity to keratinocytes
Ghahary et al. (51)	NEK, HDF, DE (HDF collagen embedded) Transwell coculture		Stratifin	Compared to HDF cultured alone, HDF in cocultures produce more MMP1 and enhance col-I digestion
Satish et al. (52)	K-foreskin, Hs68 Transwell coculture		CXCL11	CXCL11 (IP9) is induced by mechanical wounding in K CXCL11 reduces EGF-induced fibroblast motility and enhance EGF-induced keratinocytes motility
Funayama et al. (53)	NEK, keloid-K, HDF, keloid-F; Transwell coculture			Compared to NEK, keloid-K enhanced keloid-F proliferation, resistance to apoptosis (upregulation of Bcl-2) and TGF-β1 expression
Phan et al. (54)	Keloids-K, keloids-F, NEK, HDF. Transwell coculture		IGFBP-3	Compared to monocultures, HDF and keloid-F showed higher proliferation when cocultured with keloid-K. IGFBP-3 inhibition reduced keloid-F proliferation
Gron et al. (55)	NEK, HDF Coculture where NEK were grown on polycarbonate membrane coated with col-IV and added to HDF monolayers			Compared to HDF cultured alone, HDF in cocultures produce more HGF and KGF No difference in HDF proliferation
Lim et al. (56)	Keloids-K, keloids-F, NEK, HDF. Transwell coculture			HDF cocultured with keloid-K increased soluble col-I and col-III. Keloid-F cocultured with keloid-K increased both soluble and insoluble collagen
Lim et al. (57)	Keloids-K, keloids-F, HDF. Transwell coculture			Keloid-k induce proliferation HDF more than NEK
Niessen et al. (58)	Biopsies of normal and hypertrophic scars after breast surgery			High IL-1α expression at month 3 predicts normal scar, no relationship between IL-1β and TNF expression. High levels of PDGF and bFGF at 12 months correlate with hypertrophic scar
Maas-Szabowski et al. (59)	NEK, irradiated HDF, DE Coculture			Compared to HDF cultured alone, HDF in coculture expresses more KGF, IL-1α, IL-1β but less IL-8, TGF-β Compared to NEK cultured alone, NEK in col culture express more IL-1α, IL-8, bFGF, GM-CSF
Zhang et al. (60)	Skin explant culture Coculture (NEK seeded onto stratified HDF embedded on sterile nylon membrane)			Compared to HDF cultured alone, HDF have enhanced expression of epimorphin particularly beneath the keratinocyte layer
Garner (61)	NEK, HDF, Coculture			Compared to HDF cultured alone, col-I is decreased in cocultures
Ralston et al. (62)	NEK, DE Coculture			Coculture enhances matrix contraction and FN
Sato et al. (63)	NEK, HDF, DE Coculture		IL-1α	Compared to HDF cultured alone, PGE2 production is increased in cocultures via enhanced expression of COX-2 induced by IL-1α
Boxman et al. (64)	NEK, HDF K-CM			Compared to HDF cultured alone, IL-6, IL-8, production is higher and IL-1 lower in HDF exposed to NEK-conditioned medium
Chang et al. (65)	NEK, HDF, Transwell coculture			Compared to HDF cultured alone, col-I and GAG production is reduced in cocultures more so if NEK is hydrated
Lacroix et al. (66)	NEK, HDF, DE Coculture			Compared to HDF cultured alone, col-I and FN production is increased in coculture
Boxman et al. (67)	K-foreskin, HDF Coculture CM		IL-1α	Compared to HDF cultured alone, IL-1α production is increased in cocultures and K-foreskin conditioned medium
Waelti et al. (68)	NEK, irradiated HDF Coculture		IL-1β	Compared to HDF cultured alone, IL-6 production is increased in cocultures and NEK conditioned medium, effect mediated by IL-1β

*The references are reported in inverse chronological order. αSMA, alpha-smooth muscle actin; bFGF, basic fibroblast growth factor; CCD966SK, fibroblast cell line; CCL, chemokine (C-C containing) motif; CCR, receptor; for CCL chemokines; CM, conditioned medium; Col, collagen; COX2, Cyclooxygenase 2; CTGF, connective tissue growth factor; CTNNA1, Catenin Alpha 1; CXCL, chemokine (C-X-C containing)motif ligand; DE, dermis equivalent; Differentiated-K, Differentiated keratinocytes; E2, estrogen; EE, epidermal equivalent; EGF, epidermal growth factor; ENaC, Epithelial sodium channel; ENA-78, Epithelial neutrophil-activating protein 78; ET-1, endothelin-1; FAP, fibroblast activation protein; FGF, fibroblast growth factor; FN, fibronectin; Foreskin K, newborn foreskin keratinocytes; G-CSF, granulocyte colony stimulating factor; GM-CSF, granulocyte-monocyte colony stimulating factor; HaCaT, human keratinocytes immortalized cell line; HD, healthy donor; HDF, healthy donor fibroblasts; HMGB1, high mobility group box-1; Hs68, foreskin fibroblasts cell line; IGF, insulin-like growth factor; IGFBP, insulin-like growth factor binding protein; IL, interleukin; ITGA1, alpha 1 subunit of integrin receptors; K-CM, keratinocytes conditioned medium; Keloids-F, keloids fibroblasts; Keloids-K, keloids keratinocytes; KGF, keratinocyte growth factor; K-MV, Keratinocyte-derived microvesicles; LIF, leukemia inhibitory factor; MCP-1, monocyte chemotactic protein-1; M-CSF, monocyte colony stimulating factor; MMP, metalloproteinase; NEK, healthy donor keratinocytes; OPG, osteoprotegerin; OSM, oncostatin M; PAI, plasminogen activator inhibitor; PDGF, platelet-derived growth factor; PGE2, Prostaglandin E2; S100A7, psoriasin; S100A8/A9, calprotectin; S100A15, koebnerisin; SSc, Systemic sclerosis; SSc-F, SSc fibroblasts; SSc-K, SSc keratinocytes; TGF, Transforming growth factor; THBS1, Thrombospondin 1; TN-C, tenascin C; TNFα, Tumor necrosis factor α; uPA, urokinase-type plasminogen activator; UVB, ultraviolet B radiation*.

**Table 2:** *In vitro* and *ex vivo* studies on the effect of human dermal fibroblasts on keratinocytes.

**Ref**	**Type of cells or samples**	**Type of stimuli**	**Identified mediators**	**Experimental outcome**
Kumtornrut et al. (69)	NEK, HDF Coculture	Testosterone	KGF2 (FGF10)	Androgen-stimulated HDF, reduce NEK differentiation (keratins), effect mediated by FGF10
Yang et al. (70)	NEK, HDF Skin equivalent	TGF-β bFGF	KGF	TGF-β enhanced αSMA, VEGF and reduced KGF and HGF expression in HDF. bFGF reduced αSMA, but increased KGF expression in HDF. In skin equivalent, bFGF enhanced epidermal differentiation via KGF
Quan et al. (71)	NEK, HDF Skin equivalent	SDF-1		SDF-1 is expressed selectively in HDF and is hyper expressed in psoriatic skin. SDF-1 overexpression increases epidermal thickness with increased keratinocytes layers, in skin equivalent. SDF activates ERK pathway on keratinocytes.
Fernandez et al. (72)	NEK, HDF Transwell coculture Skin equivalent	Keratinocytes UVB exposure		HDF enhance NEK survival, DNA repair, and reduce apoptosis after UVB exposure by reducing caspase-3 and enhancing p53 activities
Varkey et al. (73)	NEK, superficial (S) and deep (D) HD EE S- or D-DE (cross-linked col-I–GAG matrix) Skin equivalent			In skin equivalents either engineered from S- or D-HDF, the epidermal production of IL-1α, TGFα, PDGFα was increased compared to EE alone. Only skin equivalent engineered from D-HDF showed increased epidermal production of PDGF compared to EE alone. Skin equivalent engineered either from S- or D-HDF showed increased IL-6 and KGF production compared to S- or D-DE alone. Skin equivalent showed a reduced expression of col-I and active TGFβ1 compared to DE (mainly for D-DE)
Arai et al. (23)	NEK and HDF K-CM; F-CM; EE, DE (collagen matrix) Skin equivalent			IL-1α derived by keratinocytes increase expression of PGE2 and other IL-1 inducible genes (IL6, GM-CSF and KGF) by fibroblasts (shown in skin equivalent or K-CM model of K–F interaction). Dermis derived PGE2 promote epidermis proliferation.
Sun et al. (27)	HaCaT, CCD966SK (fibroblasts cell line); 2D cultures		KGF, IL-19	KGF enhances proliferation and IL-19 production by HaCaT and IL-19 induces KGF expression in CCD966SK fibroblasts.
Canady et al. (28)	NEK, HDF, SScF, keloid fibroblasts, K-CM, F-CM skin explant		KGF, OSM	KGF is increased in keloid and SSc fibroblasts and sera. Fibroblast-derived KGF induces keratinocytes to release OSM leading to fibroblast activation (increased col-I, FAP, and migration) (results from K- or-F-CM and 2D models of interaction). In *ex vivo* skin explant confirmed that KGF increases the production of OSM, FAP, col-I
Chowdhury et al. (74)	NEK, HDF, F-CM			Compared to monolayer, coculture enhances NEK adhesion and proliferation
Yang et al. (75)	NEK, HDF, skin equivalent			Epidermal differentiation is enhanced in the absence of myo-fibroblasts. bFGF reduces αSMA expression and enhanced keratin 10 but reduced keratin 16 and TGF-β in the epidermis
Wang et al. (76)	NEK, HDF, coculture or trans-well coculture		IL-1 + TGF-β1 (HDF) HB-EGF (NHK)	In coculture, HDF enhance NEK proliferation. This effect may be due to autocrine HB-EGF effect. Keratinocyte HB-EGF expression may be induced by fibroblast-derived IL-1α and TGF-β
Kolar et al. (29)	HaCaT, NEK, Transwell coculture K- or F-CM		IL-6, IL-8, CXCL-1	F-CM and IL-6, IL-8, CXCL-1 increase the expression of keratin-8 in NEK NEK enhances the expression of IL-6, IL-8, CXCL-1,KGF, bFGF, FGF-5 in HDF
Carr et al. (77)	NEK, HDF, coculture		Stratifin (14-3-3)	Compared to monolayers, HDF enhance 14-3-3σ 1 expression in NEK
Peura et al. (78)	NEK, CRL2088-F fibroblast cell line, HDF in fibrin matrix	CRL2088-F in fibrin matrix (=Finectra)	EGF	Compared to NEK cultured alone, CRL2088-F in fibrin matrix provides better NEK viability and migration. An inhibitor of EGFR/c-Met receptor tyrosine kinases abolished keratinocyte responses
Chong et al. (79)	NEK, HDF, DE (collagen matrix) skin equivalent		PPAR α/δ	Compared to wild-type skin equivalents, PPAR α/δ-deficient fibroblasts enhanced NEK proliferation, IL-1 expression, activation of TAK1 and up-regulation of AP-1 controlled mitogenic target genes
El Ghalbzouri and Ponec (80)	NEK, HDF, F-CM skin equivalent,		Soluble factors	Compared to conditioned medium generated form epidermal equivalents or HDF, conditioned media from skin equivalents (NEK + HDF) enhanced NEK viability and differentiation, and resulted in higher deposition of laminin 5 and nidogen in the basal membrane via the release of soluble factors
Sorrell et al. (81)	NEK, HDF, S-DE, D-DE, EE, S- or D-DE (collagen matrix) skin equivalent			Compared to D-DE, S-DE resulted in higher GM-CSF/KGF ratio and enhanced IL-6 production. NEK cultured in skin equivalents with S-HDF, compared to D-HDF, showed enhanced differentiation and formation of basement membrane
Maas-Szabowski et al. (82)	HaCaT, NEK, HDF, Skin equivalent	TGF-α	IL-1α, GM-CSF, KGF (FGF7)	IL-1 epidermal derived stimulate fibroblast production of AP1-related genes, among this KGF and GM-CSF stimulate keratinocytes proliferation and secretion of IL-1. Autocrine epidermal TGFα production induce epidermal expression of the receptor for KGF and GM-CSF. HaCaT differentiation in skin equivalent is impaired, as well as IL-1 production and response to KGF and GM-CSF. This effect is due to the lack of TGFα
El Ghalbzouri et al. (83)	NEK, HDF, EE, DE (repopulated dermis matrix) skin equivalent		KGF	Complete *in vitro* generation of a differentiated epidermis requires the presence of HDF in a repopulated dermal equivalent. Fibroblast presence promotes keratinocyte proliferation, downregulates K6 and abate K16 and K17 expression. The fibroblast presence can be substituted by KGF
el-Ghalbzouri et al. (84)	NEK, HDF, EE, DE (collagen matrix) skin equivalent			As El Ghalbzouri et al. However, the expression of integrin α6β4 and of E-CAD was not dependent on HDF.
Blomme et al. (85)	NEK; HDF 2D cultures	PTHrP		PTHrP produced by K increases KGF secretion by fibroblasts
Monical and Kefalides (86)	NEK; HDF Transwell coculture			Coculture promote NEK proliferation, compared to monolayer; coculture increased protein synthesis in both cell types, compared to monolayer. Production of laminin is modulated in coculture in both cell types
Smola et al. (87)	NEK; HDF DE (irradiated or not); skin equivalent coculture			NEK proliferation increases in coculture or in skin equivalent compared to EE alone) NEK increased HDF production of IL-6; KGF, GM-CSF, collagenase compared to 2D culture Irradiation affected GM-CSF production (lower in irradiated vs. not) and collagenase production (higher in irradiated vs. not)

*The references are reported in inverse chronological order. 2D, 2-dimentions; AP-1, Activator protein 1; bFGF, basic fibroblasts growth factor; CCD966SK, human normal fibroblasts cell lines from adult; C-l, collagen I; CM, conditioned medium; CRL2088, human normal fibroblasts cell lines from foreskin; CXCL, chemokine (C-X-C containing) motif ligand; D-DE, deep dermis equivalent; DE, dermis equivalent; D-HDF= deep dermal fibroblasts; E-CAD, E-cadherin; EE, epidermal equivalent; EGF, epidermal growth factor; F, fibroblasts; FAP, fibroblast activating protein; FGF, fibroblasts growth factor; GAG, Glycosaminoglycan; GM-CSF, granulocyte-monocyte growth factor; HaCaT, human normal keratinocyte cell line from adult; HB-EGF, heparin binding EGF like growth factor; HDF, healthy donor fibroblasts; HGF, hepatocyte growth factor; IL, interleukin; K, keratinocytes; KGF, keratinocyte growth factor; NEK, normal epidermal keratinocytes; OSM, oncostatin M; PDGF, platelet-derived growth factor; PGE2, prostaglandin E2; PPAR, peroxisome proliferator-activated receptor; PTHrP, parathyroid hormone-related protein; rh, recombinant human; SCC marker, Squamous cell marker; S-DE, superficial dermis equivalent; SDF, stromal cell derived factor; S-HDF, superficial dermal fibroblasts; SMA, smooth muscle actin; TAK1, TGF activated kinase; TGF, transforming growth factor; VEGF, vascular endothelial growth factor*.

**Table 3:** Keratinocyte–fibroblast crosstalk in fibrotic pathologies.

**References**	**Pathology**	**Experimental outcome**
Dufour et al. (13)	SSc	SScF compared to HDF produce higher col-I when exposed to NEK-CM
McCoy et al. (16)	SSc	SScK compared to NEK induce higher col-I and αSMA expression by HDF
Nikitorowicz-Buniak et al. (24)	SSc	SSc epidermis expresses higher S100A9 compared to dermis and HD epidermis S100A9 enhances HDF and SScF proliferation, migration, and CTGF production
Aden et al. (36)	SSc	SSc epidermal explants produce more IL-1α resulting in enhanced gel contraction by HDF
Canady et al. (28)	SSc, keloid	SSc and keloid fibroblasts express higher levels of KGF KGF induces keratinocytes to release OSM leading to fibroblast activation
Gauglitz et al. (21)	Keloid	Keloid skin expresses lower levels of S100A7 and higher levels of COL1A1, COL1A2, COL3A1 in the dermis than HD. S100A7 decrease HDF production of COL1A1, COL1A2, COL3A1, TGF-β1, TGF-β2, TGF-β3, laminin-β2, α-SMA, and HDF proliferation
Do et al. (32)	Keloids	Keloids-K produce more IL-18 than NEK IL-18 enhances col-I, IL-6, IL-8 production by HDF
Lim et al. (37)	Keloids	Keloids-K and fibroblasts coculture produce angiogenin, OSM, VEGF, IGF-binding protein-1, OPG, and TGF-β2, and HD coculture does not
Funayama et al. (53)	Keloids	Keloid-K enhanced keloid-F proliferation, resistance to apoptosis (upregulation of Bcl-2) and TGF-β1 expression
Phan et al. (54)	Keloids	Keloid-K increased proliferation of HD and K-fibroblasts, on an IGFBP-3-dependent mechanism
Lim et al. (56)	Keloids	Keloid-K increased col-I and col-III production by HDF
Lim et al. (57)	Keloids	Keloids-k induced proliferation of HDF more than NEK
Simon et al. (31)	Hypertrophic scars	K from hypertrophic scars increase dermal matrix thickness by enhanced production of TIMP-1.
Niessen et al. (58)	Hypertrophic scars	High IL-1α expression at month 3 predicts normal scar; no relationship between IL-1β and TNF expression. High levels of PDGF and bFGF at 12 months correlate with hypertrophic scar

*The references are grouped per pathology. Additional details on culture conditions and mediators are reported in Tables 1, 2. αSMA, alpha-smooth muscle actin; bFGF, basic fibroblast growth factor; Col, collagen; CTGF, connective tissue growth factor; FGF, fibroblast growth factor; HD, healthy donor; HDF, healthy donor fibroblasts; IGF, insulin-like growth factor; IGFBP, insulin-like growth factor binding protein; IL, interleukin; Keloids-F, keloids fibroblasts; Keloids-K, keloids keratinocytes; KGF, keratinocyte growth factor; MMP, metalloproteinase; NEK, healthy donor keratinocytes; OPG, osteoprotegerin; OSM, oncostatin M;PDGF, platelet-derived growth factor; S100A7, psoriasin; S100A8/A9, calprotectin; SSc, Systemic sclerosis; SSc-F, SSc fibroblasts; SSc-K, SSc keratinocytes; TGF, Transforming growth factor; THBS1, Thrombospondin 1; TIMP, tissue inhibitor of metalloproteinases; TNF, Tumor necrosis factor; VEGF, vascular endothelial growth factor*.

Figures were generated using Biorender.com and Inkscape (http://www.inkscape.org/).

## Results

### Literature Search

**Figure 1:**
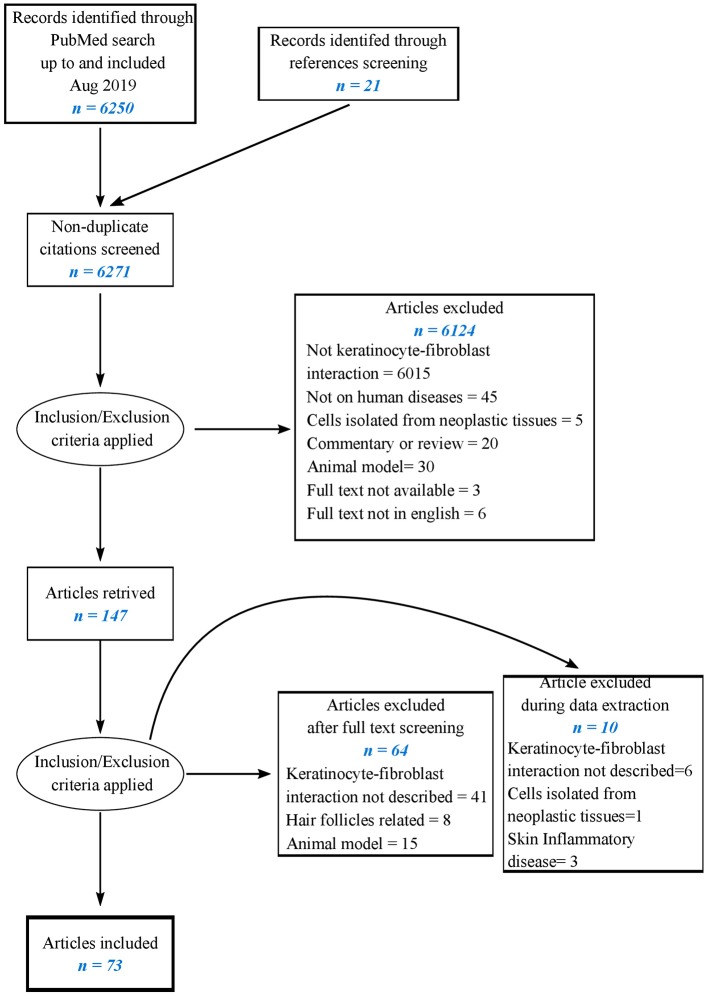
Flow chart of the literature-searching strategy.

The literature search resulted in 6,250 hits from PubMed and 21 from reference screening. After the screening of titles, abstracts, and full texts, 73 articles were included in the present review as reported in the flowchart (Figure 1). Six studies examined simultaneously the reciprocal effect of keratinocytes on fibroblasts and of fibroblasts on keratinocytes. Fifty-six papers explored the effects of keratinocytes on fibroblasts, 19 of which investigating soluble factors of inflammation and growth factors, 17 reporting cell proliferation, differentiation, and migration, extracellular matrix components, and turnover, 10 reporting responses to soluble factors and physical stress, 14 referring to specific skin pathologies including SSc, keloids, hypertrophic scars.

Twenty-three studies investigated the effects of fibroblasts on keratinocytes, 14 of which investigated keratinocyte proliferation, differentiation, activation, survival, and adhesion; two investigated keratinocyte production of inflammatory mediators. Six papers focused on responses to soluble factors or altered expression of transcription factors. Two papers investigating the effect of fibroblasts on keratinocytes focused on pathological conditions (SSc and keloids).

### Experimental Models Used to Assess the Crosstalk Between Keratinocytes and Fibroblasts

**Figure 2:**
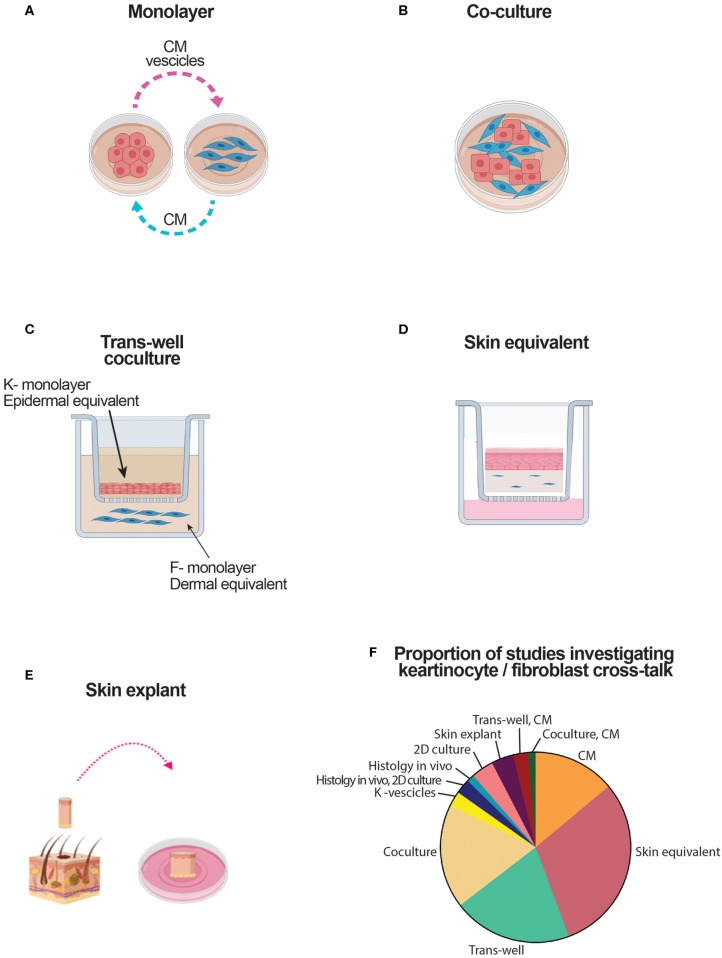
Schematic representation of culture systems used to assess the crosstalk between keratinocytes and fibroblasts. CM, conditioned medium; DE, dermal equivalent; EE, epidermal equivalent; F, fibroblast; K, keratinocyte. **(A)** Culture based on the use of medium conditioned by one type of cell cultured in monolayer to modulate the response of the other cell type. **(B)** Culture based on a mix of keratinocytes and fibroblasts. **(C)** Culture based on the use of transwells. Keratinocytes either in monolayer, either in dermal equivalents are in the top well. Fibroblasts, either in monolayer either in dermal equivalent are in the bottom well. Soluble mediators cross the semipermeable transwell membrane. **(D)** Skin equivalent generated at the air liquid interphase. **(E)** Organotypic full skin culture obtained by skin biopsy. **(F)** Proportion of the studies addressing keratinocyte-fibroblast crosstalk which results are reviewed here.

The experimental models used to assess the crosstalk between keratinocytes and fibroblasts are schematically reproduced in Figure 2, and analytically reported in Tables 1, 2. Many papers combined two or more experimental models. The simplest and straightforward experimental approach used in 11 papers was based on the use of the conditioned medium (CM) to be transferred from a cell type to the other (Figure 2A). A potential drawback may be related to differential media requirements for optimal survival, proliferation, and differentiation of keratinocytes and fibroblasts. Two papers have used centrifugation of CM to enrich for keratinocyte microvesicles or exosomes to be tested on fibroblasts (20, 38). Physical coculture of keratinocytes with fibroblasts was used in 14 papers (Figure 2B). The Transwell technology has been adapted to assess many different cell combinations for a total of 16 papers. Thus, keratinocytes put in the upper well-could have been cultured in monolayers or could be grown to reach stratification and differentiation to become epidermal equivalents (EE) (Figure 2C). Similarly, fibroblasts put in the lower well could have been grown in monolayers adherent to plastic or embedded in a matrix, thus generating a dermal equivalent (DE) (Figure 2C). Skin equivalents generated in cultures based on air/liquid interphase have been used in 24 papers (Figure 2D). Finally, full skin organotypic culture has been used in three papers (Figure 2E) (13, 28, 60). The proportion of studies using these culture methods is reported in Figure 2F.

### Effects of Keratinocytes on Fibroblasts

#### Effects of Keratinocytes on the Production by Fibroblast of Soluble Factors of Inflammation and Growth Factors

Enhanced interleukin (IL)-6 production by fibroblasts submitted to the influence of keratinocytes was robustly identified in eight studies of eight in healthy donors (HD) (13, 20, 23, 26, 32, 50, 64, 68). Enhanced production of IL-8 was identified in six studies of seven in HD (13, 18, 26, 29, 32, 64) with a decreased IL-8 production in one of seven (59). Enhanced production of monocyte chemotactic protein (MCP)-1 was identified in three studies of three (13, 18, 50). Enhanced production of cyclooxygenase (COX)2 was identified in two studies of two (23, 50). Enhanced production of IL-1 (IL-1α, or IL-1β, or IL-1 with no specification) was identified in four studies of six (26, 29, 59, 67), with a decreased IL-1 production in two of six (41, 64). Enhanced production of prostaglandin E2 (PGE2) (63), chemokine (C-X-C motif) ligand 1 (CXCL1) (29), and tumor necrosis factor (TNF)-α (41) by fibroblasts was identified in single studies. Enhanced keratinocyte growth factor (KGF), also known as fibroblast growth factor (FGF) 7, production by fibroblasts submitted to the influence of keratinocytes was identified in four studies of five in HD (27, 29, 55, 59), with a decreased KGF production in one of five (23). Enhanced production of granulocyte-colony-stimulating factor (G-CSF) was identified in two of two in HD (17, 50), while transforming growth factor (TGF)-β was found to be decreased in two of two studies (42, 59). For connective tissue growth factor (CTGF), one study reported enhanced (26), and on other study decreased, production (35) by HD fibroblasts. Enhanced production of vascular endothelial growth factor (VEGF)-1, platelet-derived growth factor (PDGF)-1, hepatocyte growth factor (HGF), basic FGF (bFGF), monocyte (M)-CSF, granulocyte monocyte (GM)-CSF, and epimorphin were all found in single studies (17, 23, 26, 55, 60).

#### Effects of Keratinocytes on Fibroblast Proliferation, Differentiation, Migration, Extracellular Matrix Components, and Turnover

Type-I collagen (col-I) production by fibroblasts submitted to the influence of keratinocytes was found enhanced in four studies (13, 16, 32, 66) and decreased in eight of 12 studies in HD (26, 35, 39, 41, 43, 50, 61, 65). Fibronectin (FN) production was reported to be enhanced in five of six studies (33, 44, 45, 62, 66) and decreased in one of six (35). Other ECM components including tenascin, versican, lumican, and thrombospondin were variably reported to be increased in two or decreased in one of three studies (20, 33, 49). The production of matrix metalloproteinases (MMP) including MMP-1, MMP-2, MMP-3, MMP-8, MMP-9, and MMP-12 when investigated was always found to be increased in fibroblast under the influence of keratinocytes for a total of 12 studies (13, 18, 20, 34, 35, 38, 40, 44, 46–48, 51). The production of tissue inhibitor of metalloproteinases (TIMP)-1, TIMP-2, and TIMP-3 was variably reported to be increased in two (34, 48) or decreased in one of three studies (35). Decreased production of cathepsins was reported in one paper (50). The production of plasminogen activator inhibitor (PAI) was variably reported to be increased in two (44, 50) and decreased in one of three studies (35). Uroplasminogen (uPA) was increased in two of two papers (35, 40). Decreased proliferation of HD fibroblasts was reported in two (45, 57) and unchanged in one of three (55). Enhanced fibroblast migration was reported in three of three papers (20, 25, 27). Four papers reported enhanced HD fibroblast proliferation mediated by keratinocyte produced S100A9 (24), or by UVB-exposed keratinocytes cultured in estradiol (30) or enhanced migration (25) mediated by microvesicles released by keratinocytes (20). Cell adhesion and cadherin expression were found increased in two papers each (20, 44), and gel contraction increased in three of three (20, 49, 62). The expression of α-smooth muscle actin (αSMA) was variably reported to be increased in two (49, 50) or decreased in one of three studies (35).

#### Effect of Soluble Factors and Physical Stress Acting on Keratinocytes for Their Influence on Fibroblasts

Within the context of wound healing, the effects on fibroblasts of dehydration or hyper-hydration of partially stratified keratinocytes was investigated in six papers (15, 19, 22, 34, 52). Robustly, five of them examining col-I production reported enhanced col-I production when keratinocytes were dehydrated compared to their normally hydrated counterpart. When tested, consistently with the results on col-I, αSMA expression was increased by dehydration in three of three papers (15, 19, 22). One of these papers focused on MMPs and TIMP-1 showing that hydration enhances MMP1, MMP8, and MMP13 and decreases TIMP-1 production (34). One paper reported that hyper-hydration of partially stratified keratinocytes enhances the production of KGF by fibroblasts (34).

Physical stimuli were investigated in two papers. One focusing on keratinocyte exposure to UVB in the presence of estradiol (E2) reported that when exposed, fibroblasts responded by enhanced proliferation and EGF and lumican production (30). Another paper reported that keratinocytes exposed to micro-particles enhanced the fibroblast production of IL-1β, IL-6, TNF-α, PGE2, MMP-1, and MMP2 (14).

Keratinocytes primed with IL-17A were reported to enhance fibroblast production of IL-6, IL-8, MCP-1, and MMP-1 (13). Keratinocytes primed with IL-22 jointly with TNF-α were reported to enhance fibroblast production of IL-8, MCP-1, and MMP-1 when compared to keratinocytes unprimed or primed with IL-22 or TNF-α alone (18).

#### Specific Skin Pathologies (Table 3)

Five papers investigated the influence of keratinocytes on fibroblasts in SSc (13, 16, 24, 28, 36). Two reported increased col-I production compared to HD (16, 28), one of them indicating a TGF-β-independent enhancement (16), one reported enhanced IL-1-dependent gel contraction in which TGF-β and endothelin (ET)-1 were needed to observe gel contraction (36). One paper reported enhanced CTGF production, enhanced fibroblast migration, and proliferation with a role of S100A9 (24). SSc fibroblasts were reported to respond with higher production of col-I, similar production of MMP-1 with an increased ratio col-I over MMP-1, suggestive of decreased ECM turnover (13).

Eight papers investigated the influence of keratinocytes on fibroblasts in keloids. Three reported enhanced fibroblast proliferation (53, 54, 57) of which one also enhanced TGF-β production (53). One paper pointed to a reduced expression of S100A7 and S100A15, which were suggested to act as inhibitors (21); one concentrated on enhanced col-I production (56). A single paper reported enhanced production of TGF-β, oncostatin M (OSM), fibroblast-activating protein (FAP), αSMA, and laminin, compared to HD (37). Enhanced production of KGF by keloid keratinocytes resulting in enhanced release by fibroblasts of OSM, and col I was reported in one paper (28). One paper reported enhanced IL-18 production by keratinocytes resulting in enhanced production of IL-6, IL-8, and col-I by fibroblasts (32).

Two papers investigating the influence of keratinocytes on fibroblasts in hypertrophic scars reported an increase in matrix thickness, PDGF, and bFGF production compared to HD (31, 58).

### Effects of Fibroblasts on Keratinocytes

#### Effect of Fibroblasts on Keratinocyte Proliferation, Survival, Adhesion, Keratin Expression

The effect of fibroblasts on keratinocyte proliferation was investigated in nine papers and reported to be increased in all of them (72, 74, 76, 82–84, 86, 87). Keratinocyte survival was reported to be enhanced in the presence of fibroblasts for reduced apoptosis, reduced expression of Bcl2, and enhanced expression of p53 (72, 78, 80). Keratinocyte adhesion and cadherin expression were reported to be enhanced in the presence of fibroblasts in two papers (72, 74). Keratinocyte differentiation was robustly reported to be enhanced in the presence of fibroblasts in four of four papers (73, 80–82), two of which specifically attributed this effect to fibroblasts from papillary compared to superficial dermis (73, 81). The influence of fibroblasts on keratin expression by keratinocytes was studied in two papers, one reporting enhanced expression of keratin 8 (29) and two others a reduced expression of keratins 6, 16, and 17 (80, 83). Fibroblasts were reported to enhance the deposition of basal membrane components by keratinocytes in three papers (73, 80, 81).

#### Effect of Soluble Factors or Altered Expression of Transcription Factors in Fibroblasts for Their Influence on Keratinocytes

Fibroblasts exposed to bFGF were reported to enhance keratinocyte differentiation in one study (70) and to reduce keratinocyte production of TGF-β in another study (75). Fibroblasts exposed to stromal cell-derived factor (SDF)-1 were reported to enhance keratinocyte proliferation and stratification in one study (71). Fibroblasts exposed to testosterone were reported to decrease keratinocyte differentiation in one study (69). Fibroblasts with inhibited expression of both peroxisome proliferator-activated receptor (PPAR)α and PPARδ were reported to enhance keratinocyte proliferation and their production of IL-1 and activator protein (AP)-1-targeted genes in one paper (79).

#### Specific Skin Pathologies (Table 3)

Fibroblasts from keloids and SSc were reported to enhance the production of oncostatin M (OSM) by HD keratinocytes in one paper (28).

### Soluble Mediators of Inflammation Influencing the Crosstalk of Keratinocytes With Fibroblasts

Among the soluble mediators of inflammation produced by keratinocytes affecting fibroblast responses, IL-1 is robustly reported to be a relevant keratinocyte-derived mediator inducing fibroblast activation in eight of eight papers addressing this aspect (13, 17, 23, 36, 63–65, 82). Conversely, three of three papers reported that fibroblasts regulate epidermal homeostasis (proliferation and differentiation) through the secretion of KGF (28, 70, 83). Keratinocyte production of TGF-β by itself or in association with other mediators including IL-1 and ET-1 was reported to enhance col-I production by fibroblasts in three of four papers (13, 36, 49). The role of keratinocyte-derived stratifin, also known under the name 14.3.3 sigma, has been extensively investigated by one group that showed its role in enhanced MMPs and reduced col-I production by fibroblasts (33, 38, 39, 44, 45, 47, 51). Further, in the presence of fibroblasts, the same group showed enhanced stratifin production by keratinocytes (77). Keratinocytes were shown to produce fibronectin resulting in enhanced fibroblast migration (25). Keratinocytes were reported to produce IL-19, which resulted in enhanced KGF production by fibroblasts. In its turn, KGF enhanced the IL-19 production by keratinocytes. Chemokine (C-C motif) ligand (CCL)26 (eotaxin-3) production by keratinocytes was reported to enhance fibroblast proliferation and motility (27). High mobility group box 1 (HMGB1) production by keratinocytes was reported to enhance fibroblast activation and αSMA expression (15). One study reported that parathyroid hormone-related protein (PTHrP) released by keratinocytes enhanced the production of KGF by fibroblasts (85). Finally, two studies reported that keratinocyte production of vesicles (whether microvesicles or exosomes) enhanced fibroblast activation with higher production of MMPs and a number of other mediators detected by microarrays (20, 38, 45).

#### Specific Skin Pathologies

In SSc, one paper reported enhanced col-I production induced by keratinocytes in a TGF-β-independent fashion (16), while a role for TGF-β was reported in two (13, 36). Expression of calprotectin, also known as S100A8/A9, was reported to be increased in keratinocytes from hypertrophic scars and SSc, resulting in enhanced fibroblast production of col-I and CTGF in two studies (19, 24). Psoriasin, also known as S100A7, was reported to be decreased in keratinocytes from keloids, a finding associated with increased col-I production by fibroblasts (21). Single papers have addressed the role of several other mediators. Collectively, the production by keratinocytes of IL-18 in keloids (32), PGE2 in dehydration (22), reduced TIMP production in hypertrophic scars (31), was associated with enhanced col-I production.

## Discussion

Our systematic review has retrieved 73 published papers investigating the interplay between keratinocytes and fibroblasts. Our main aim was to focus on fibrosis. While only 14 papers specifically aimed at skin fibrotic disorders, many focused on wound healing, which is a physiological condition considered to have several analogies with skin fibrosis, at least during the initial proliferative and synthetic phase (1). In this respect, it has to be underlined that the relatively little number of papers dedicated to this topic reflects, at least in part, the complexity of experimental settings needed to investigate the interactions between these two cell types with different requirements for optimal *in vitro* survival. This is particularly true for keratinocytes, which may undergo proliferation and differentiation under specific and mutually exclusive culture conditions. Not unexpectedly, compared to their undifferentiated counterpart, keratinocytes undergoing differentiation synthetize a distinct panel of proteins and soluble mediators and react differentially to exogenous stimuli, including those potentially provided by fibroblasts. For instance, a number of papers investigating the effect of keratinocytes on fibroblasts have used culture supernatants as effectors on fibroblasts. Furthermore, the supernatants may have been generated from non-primary keratinocyte cell lines, undifferentiated primary keratinocyte lines, and, in some instances, differentiated and stratified keratinocytes. Likely, the most physiologically relevant approaches to address the cross-talk between keratinocytes with fibroblasts were based on the use of epidermal equivalents or skin equivalents of full skin approaches. This information is provided in Tables 1, 2, and Figure 2F. However, as limitation of our review, we have not weighted the relevance of the reported results based on the experimental assay used. Of particular importance to critically apprise the mutual relationship between keratinocytes and fibroblasts is the role of the basement membrane, which separates, holding together, the epidermis and dermis *in vivo* (88). In this respect, the papers specifically studying the structure and composition of the *in vitro*-generated basement membrane acquire additional value (73, 80, 81). The experimental settings leading to the results here reviewed were mostly based on the use of conditioned medium, skin equivalents, transwells, and cocultures each contributing to about one fifth of the total. Additional approaches took advantage on combinations of methods, histology on skin tissues, and more demanding skin explants. Given the existence of a basement membrane separating the epidermis from dermis, cell-to-cell contact effects between keratinocytes and fibroblasts could have limited physiological relevance. An additional point to consider is the possibility that dermal fibroblasts adapting to *in vitro* culture growth may lose some of their tissue-specific characteristics, then impacting on their effects on keratinocytes.

Notwithstanding these considerations, the wealth of retrieved papers clearly highlights the interest in the problematics of keratinocyte to fibroblast crosstalk and the capacity of these cell types to mutually influence each other. The majority of the retrieved papers investigated how keratinocytes interact with fibroblasts in the context of wound healing, using keratinocytes and fibroblasts generated from healthy individuals, with 14 papers investigating how this interaction is modified and characteristic of pathologic conditions. Overall, a large agreement characterizes the results indicating that also in homeostatic conditions, the crosstalk between keratinocytes and fibroblasts has an impact on both cell types and ultimately on the structure of both epidermis and dermis. However, the outcome of the interactions and the factors contributing to the crosstalk were heterogeneously investigated, and in some cases, the reported results were inconsistent.

**Figure 3:**
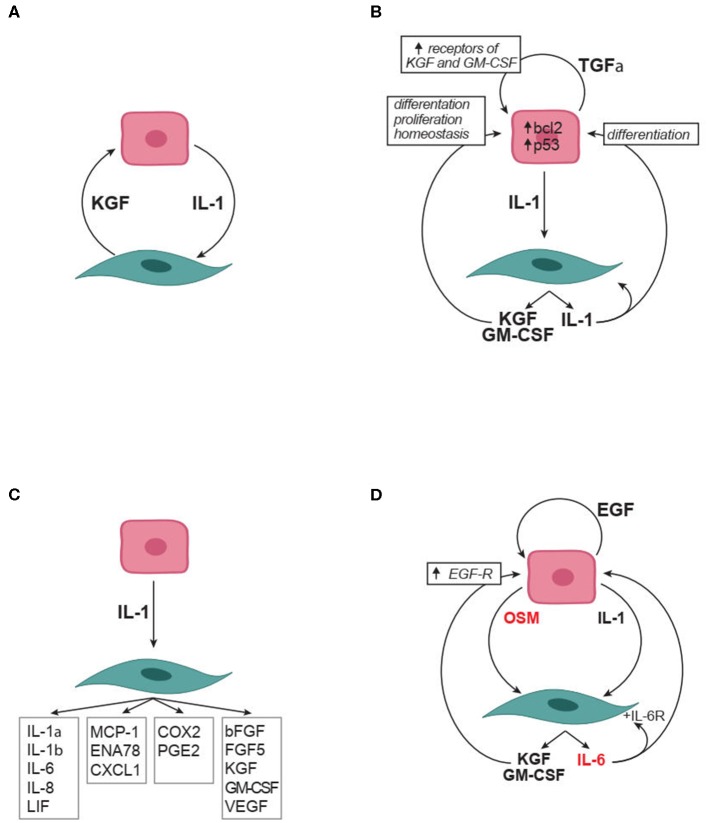
Soluble factors in the crosstalk between keratinocytes and fibroblasts. **(A)** IL-1 and KGF are soluble mediators robustly identified as involved in the crosstalk. **(B)** Autocrine and paracrine effects relevant to IL-1 and KGF role in the crosstalk. **(C)** The many effects of keratinocyte produced IL-1 on fibroblasts. **(D)** Synergistic effect of IL-1 with IL-6 family members (in red) on the crosstalk. Arrowheads indicate enhancement. bFGF, basic fibroblast growth factor; COX2, Cyclooxygenase 2; CXCL, chemokine containing the CXC motif; EGF, epidermal growth factor which comprises multiple mediators including transforming growth factor-α, amphiregulin, heparin binding-EGF, and epiregulin; ENA-78, Epithelial neutrophil-activating protein 78; FGF, fibroblast growth factor; GM-CSF, granulocyte-monocyte colony stimulating factor IL, interleukin; KGF, keratinocyte growth factor (also known as FGF7); LIF, leukemia inhibitory factor; MCP-1, monocyte chemotactic protein-1; OSM, oncostatin M;PGE2, Prostaglandin E2; TGF, Transforming growth factor; VEGF, vascular endothelial growth factor.

Strong evidence supports a role for keratinocyte-produced IL-1 in inducing fibroblast production of KGF, GM-CSF, TGFα, IL-6, IL-8, IL-1, the expression of COX2 and PGE2 production (Figures 3A–D). In its turn, KGF, GM-CSF, and PGE2 promote keratinocyte proliferation and favor proper keratinocyte differentiation (Figures 3A,B,D). Simultaneously, TGFα enhances the expression on keratinocytes of both receptors for KGF (FGFR2b) and GM-CSF (GM-CSF-R), thus favoring keratinocyte responses to these ligands (Figure 3B). Furthermore, PGE2, IL-6, GM-CSF, and KGF produced by fibroblasts enhance IL-1 production by keratinocytes, thus promoting a positive forward amplification loop (Figure 3D). Not last, the autocrine production of IL-1 by fibroblasts may amplify fibroblast production of several mediators including KGF and GM-CSF (Figures 3A,D). It is of interest to notice that the circuitries here reported and highlighted in Figure 3 all propose positive feedforward effects. It is very unlikely that this reflects the reality since biological systems have inbuilt physiological modulators and inhibitors. Thus, further homeostatic factors and inhibitory mechanisms important in the crosstalk between keratinocytes and fibroblasts likely will be identified in future work. It is, however, true that feed-forward mechanisms may participate in pathological processes.

**Figure 4:**
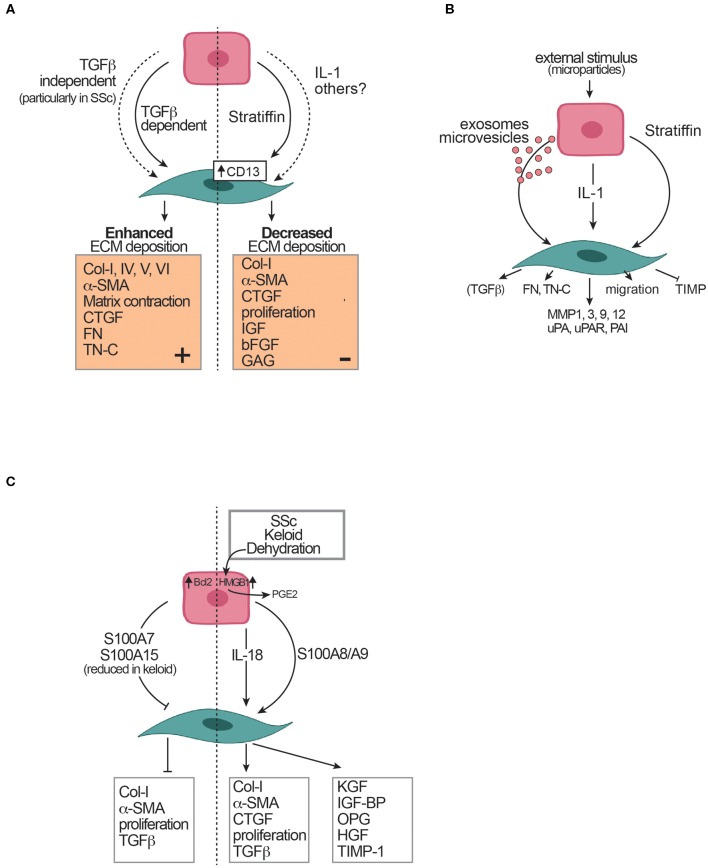
Effects of keratinocytes on fibroblasts and extracellular matrix (ECM). **(A)** Controversial effects of keratinocytes on ECM deposition. **(B)** Mediators of keratinocyte effects on fibroblasts. **(C)** Skin pathological conditions and their effects on the crosstalk between keratinocytes and fibroblasts. The dotted vertical line separates controversial evidence. Arrowheads indicate enhancement. Blunted heads indicate inhibition. αSMA, alpha-smooth muscle actin; Bcl2, B-cell lymphoma 2; bFGF, basic fibroblast growth factor; Col, collagen; CTGF, connective tissue growth factor; FN, fibronectin; HGF, hepatocyte growth factor; HMGB1, high mobility group box-1; IGF, insulin-like growth factor; IGF-BP, insulin-like growth factor binding protein; KGF, keratinocyte growth factor; MMP, metalloproteinase; OPG, osteoprotegerin; OSM, oncostatin M; PAI, plasminogen activator inhibitor; PDGF, platelet-derived growth factor; PGE2, Prostaglandin E2; S100A7, psoriasin; S100A8/A9, calprotectin; S100A15,koebnerisin; SSc, Systemic sclerosis; SSc-F, SSc fibroblasts; SSc-K, SSc keratinocytes; TGF, Transforming growth factor; TIMP, tissue inhibitor of MMP; TN-C, tenascin C; TNFα, Tumor necrosis factor α; uPA, urokinase-type plasminogen activator; uPAR, urokinase-type plasminogen activator receptor.

Several papers retrieved in our systematic review address the effect of keratinocytes on ECM component production by fibroblasts. Controversial are the results reported on collagen deposition and other ECM components. Thus, while a majority of studies (eight of 12) demonstrate an inhibitory role of keratinocytes, four of 12 papers reported an enhancing effect of keratinocytes on collagen production (Figure 4A). One paper proposed for the enhanced production of collagen a TGF-β-independent keratinocyte contribution (16), the others via TGF-β. It is difficult to reconcile these contradictory results; however, substantial differences in the experimental settings including the culture medium composition, the differentiation status of keratinocytes, as well as the methods used to quantify collagen may explain the differences observed. For future studies, it will be important to standardize further the experimental settings to allow robust comparisons across results.

Consistent with the majority of reports showing a decreased production of collagen by fibroblasts under the influence of keratinocytes, the fibroblast production of TGF-β and CTGF was reported to be downregulated by keratinocytes. However, keratinocytes form keloids, hypertrophic scars, and SSc that distinctly showed enhanced expression of the alarmin S100A8/A9, which directly favored collagen and CTGF production and αSMA expression by fibroblasts, thus pointing to pathology-associated differences compared to controls (Figure 4C). Further, among the states of perturbed homeostasis, keratinocyte dehydration was frequently investigated and consistently found to favor profibrotic responses in fibroblasts (Figure 4C). Finally, decreased expression of S100A7 and S100A15 in keloids may mechanistically be linked to enhanced collagen production since they were reported to be inhibitory (21).

In contrast with the inconsistent results reported on collagen production, there was a strong agreement among reports showing that keratinocytes enhance MMP production by fibroblasts (Figure 4B). One paper reported, in addition, a decreased production of TIMP by fibroblasts under the influence of keratinocytes. Overall, the picture that emerges from these studies supports a model in which keratinocytes favor ECM turnover by favoring MMP over a concomitant decreased or alternatively increased collagen production by fibroblasts. Of interest, one paper exploring this issue reports that in SSc compared to healthy controls, the ratio of collagen over MMP-1 is distinctly in favor of enhanced deposition, such as an effect not being present in HD (13). IL-1 stands out among the soluble factors produced by keratinocytes involved in the enhanced production of MMPs by fibroblasts (Figure 4B). In addition, one group has devoted enormous attention to the role of stratifin expressed by keratinocytes in inducing MMP and decreasing collagen, CTGF, insulin-like growth factor (IGF), bFGF, glycosaminoglycan (GAG) production as well as the expression of αSMA by fibroblasts (Figures 4A,B). Of further interest, the possibility that exosomes released by keratinocytes may be, at least in part, mediators of this effect (38) and that soluble factors released by fibroblasts may modulate stratifin production by keratinocytes (77).

**Figure 5:**
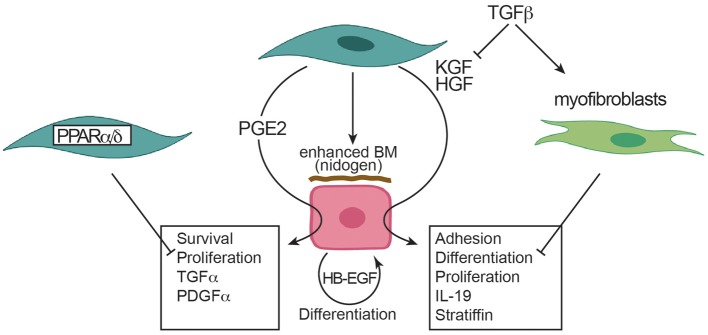
Effects of fibroblasts on keratinocytes. Arrowheads indicate enhancement. Blunted heads indicate inhibition. BM, basal membrane; HB-EGF, heparin binding EGF like growth factor; HGF, hepatocyte growth factor; IL, interleukin; KGF, keratinocyte growth factor; PDGF, platelet derived growth factor; PGE2, prostaglandin E2; PPAR, peroxisome proliferator-activated receptor; TGF, transforming growth factor.

Concerning the influence of fibroblasts on keratinocytes, the literature provides solid and consistent evidence that, in the presence of fibroblasts, keratinocytes show enhanced proliferation, reduced apoptosis, physiological differentiation, enhanced basement membrane deposition (Figure 5). These effects are mediated mostly by KGF (Figure 3A). Other important soluble factors are HGF and PGE2 (Figure 5). Of interest, TGF-β and myofibroblasts exert an inhibitory role particularly on keratinocyte differentiation and proliferation. Further, the deficiency of PPARα and PPARδ in fibroblasts promotes keratinocyte proliferation and, among others, enhanced IL-1 production (Figure 5). However, only two papers provided data on the effect of fibroblasts on keratinocytes in fibrotic disorders (28, 37) showing stronger effects of fibroblasts from fibrotic disorders. The paucity of studies exploring this topic most likely may be explained by the fact that keratinocytes are not currently integrated in physiopathological models of fibrosis development. However, the recent documentation of altered keratinocyte differentiation and inflammatory response in skin fibrosis begs the question whether these abnormalities are primary or secondary to dermal fibrosis. Thus, at the moment, it remains an interesting area of research to investigate whether fibroblasts generated form SSc may affect keratinocyte behavior.

## Conclusions

Evidence generated in recent years and reviewed here strengthen a role for keratinocytes in participating in dermal fibrosis. Whether this is a modulatory role rather than an initiation role remains to be established firmly. Murine models support the possibility that keratinocytes may indeed instruct fibroblast to enhance ECM deposition. For instance, Brakebusch et al., observed the development of dermal fibrosis in a mouse deficient for the β1 integrin subunit in keratinocytes (89). Similarly, the keratinocyte-specific genetic deletion of Friend leukemia virus integration 1 (Fli1) induced in mice a SSc-like phenotype with skin, esophageal, and lung involvement (90). To further strengthen this point, a system level analysis based on consensus clustering of genes expressed in human SSc skin revealed that keratinocytes make major connections with the inflammation network, thus highlighting their role in SSc (91).

Further work is required to better understand the reciprocal role of keratinocytes and fibroblasts and their interactions at initiation and stabilization of skin fibrosis. In this respect, novel sophisticated technical approaches may provide important new information. For instance, the generation of human skin equivalents where keratinocytes, dermal fibroblasts, and endothelial cells are grown on a biological scaffold and perfused at physiological pressure have very recently been shown to respond to fibrotic stimuli (92). Thus, vascularized skin equivalents can replicate key features of fibrotic skin and may serve as a platform to better understand the interplay between different cell types including keratinocytes and fibroblasts in pathophysiologically relevant human setting. Skin generated from stem cells and human organoids or humanized mouse models may provide additional tools for approaching similar questions (93). A complementary *ex vivo* approach would be the use of precision cut slices of healthy and diseased human skin, which would recapitulate the organ architecture then analyzed by advanced imaging techniques (94). Further, single-cell mRNA studies from cells freshly obtained from healthy and diseased skin will expand our knowledge, particularly comparing wound healing to fibrotic skin disoders. These approaches will possibly capture the subtle mechanisms involved in rapid termination of ECM deposition, which very likely distinguish physiological reparative processes from pathological fibrosis. This may lead to the development of novel therapeutic strategies.

## Author Contributions

BR performed the systematic review of the literature. BR, NB, and CC drafted the manuscript, reviewed its contents, and approved its final version.

## Conflict of Interest

The authors declare that the research was conducted in the absence of any commercial or financial relationships that could be construed as a potential conflict of interest.
